
JOURNAL INFORMATION
==============================
NLM Title Abbreviation: Inter Econ
Journal ID: Inter Econ
NLM Journal ID: 101086399

Inter Economics

ISSN: 0020-5346

ARTICLE INFORMATION
==============================
PMCID: PMC7584859
DOI: 10.1007/s10272-020-0915-z
Article ID: 915
Article version: 1
Subjects: Forum

Reforming EU Fiscal Rules: More Leeway, Investment Orientation and Democratic Coordination

Truger Achim achim.truger@uni-due.de

grid.5718.b 0000 0001 2187 5445 Institute for Socio-Economics, University Duisburg-Essen, Forsthausweg 2, 47057 Duisburg, Germany
Electronic publication date: 2020 Oct 24
Print publication date: 2020
Volume: 55
Issue: 5
First page: 277
Last page: 281
Copyright: © The Author(s) 2020
License: Open Access: This article is distributed under the terms of the Creative Commons Attribution 4.0 International License (https://creativecommons.org/licenses/by/4.0/).
Open Access funding provided by ZBW — Leibniz Information Centre for Economics.
License URL: https://creativecommons.org/licenses/by/4.0/

issue-copyright-statement: © The Author(s) 2020

==============================

In February 2020, the European Commission started its Economic Governance Review, in which EU fiscal rules obviously play a prominent part. The unprecedented economic crisis caused by the COVID-19 pandemic has understandably shifted attention away from the fiscal framework. In March, the European Council activated the general escape clause of the Stability and Growth Pact (SGP) and since then the debate focused on EU level emergency measures to overcome the crisis. After a somewhat shaky start, the EU responded with collective financial support. Safety nets worth €540 billion were approved in April, with credit lines for all member states. Encouraged by the joint initiative of Emmanuel Macron and Angela Merkel, a one-off recovery plan for Europe with €750 billion was approved – part of which is earmarked in particular as direct support for heavily affected states. The funds are to be raised through borrowing in the name of the EU, to be serviced through the EU budget – partly through its to-be-created own tax revenues. The agreement reached at the EU summit in July 2020 represents a major breakthrough on the road to reforming the EU's fiscal governance, which had largely come to a standstill before the crisis.

EU fiscal rules, however, still urgently need a reform in order to strengthen the role of fiscal policy. First, the abolition of national monetary policy in the euro area means that fiscal policy must play a much larger role in stabilising national economies. The ECB has to orient its interest rate policy by the Economic and Monetary Union (EMU) average and therefore is unable to respond to specific economic circumstances in individual countries. In the absence of fiscal countermeasures at the national level, this threatens to create persistent boom-bust cycles capable of endangering the stability of the EMU. Second, particularly during periods of crisis, fiscal policy must support monetary policy, whose stabilisation possibilities are restricted at the zero lower bound for interest rates. Third, as recent empirical results for the fiscal multiplier show, fiscal policy is much more effective macroeconomically than previously assumed, especially in periods of crisis. Fourth, fiscal policy must enable strong long-term productivity growth through high and consistent public investment in traditional and ecological infrastructure and in education and research.

## The threat of further austerity

The crucial importance of fiscal policy for macroeconomic development in the euro area is illustrated by the fact that the acute economic crisis in the countries of the European periphery could – at least for the time being – only be overcome by relaxing fiscal rules and thus by a much less restrictive fiscal policy stance. After the tightening of European fiscal rules (six-pack, Fiscal Compact, two-pack) had led to a strict austerity policy in these countries (Seikel, 2016), the EU Commission under Jean-Claude Juncker interpreted and applied the rules in a more relaxed way (European Commission, 2015; European Council, 2015). This, together with the ECB's willingness, declared in 2012, to provide guarantees for the government bonds of the affected countries, finally paved the way for an economic recovery.

Figure 1 uses the EMU's and the average of four crisis countries' (Greece, Italy, Portugal and Spain) structural budget balance[1] to show the orientation of fiscal policy. If structural balances rise (decrease), this signals a restrictive (expansive) fiscal policy. With the austerity policy in place between 2010 and 2013, the structural budget balance was reduced by more than 6% of GDP in only three years, dragging the periphery into a severe double-dip recession. The initially weak and then somewhat stronger upswing since 2014 was driven by domestic demand and coincides with a perceptible relaxation of the consolidation policy: in 2014, fiscal policy switched to a neutral stance. Brussels' budgetary surveillance ultimately tolerated the deterioration of the structural budget balance. This was partly also a consequence of the reinterpretation of the rules of the SGP.

Another important aspect relates to the development of public investment (Figure 2): from 2009 to 2012, during the

1 The structural budget balance is the government budget balance adjusted by the European Commission for cyclical influences and one-off effects. The EU Commission has published both balances since 2010 (see Mourre et al., 2014).

Figure 1

**General government budget balance and structural balance, EMU and EMU periphery, 2007-2021**

in % of (potential) GDP

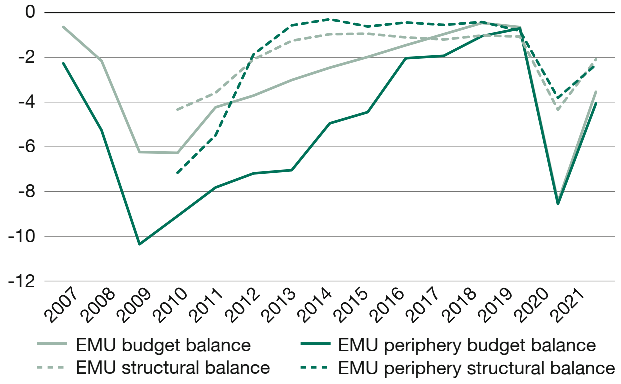

Note: The EMU periphery refers to Greece, Italy, Portugal and Spain; unweighted arithmetic average.

Source: European Commission (2020); author's calculations.

Figure 2

**General government net capital formation, EMU and EMU periphery, 2007-2021**

in % of GDP

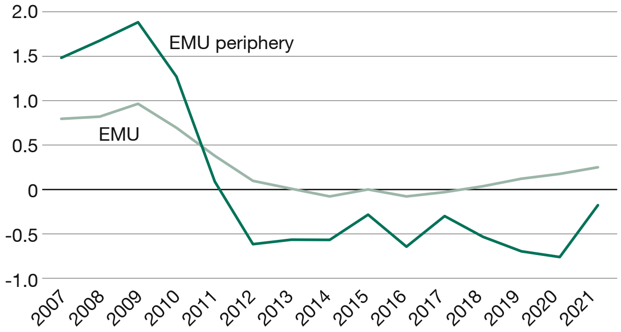

Note: The EMU periphery refers to Greece, Italy, Portugal and Spain; unweighted arithmetic average.

Source: European Commission (2020); author's calculations.

acute austerity phase, net public investment in the periphery dropped by more than 2% of GDP. The multiplier of public investment is particularly high and reductions are therefore particularly harmful (Gechert, 2015). Nevertheless, investment was severely affected by cuts because it is usually a non-compulsory task and therefore the first target of austerity. It was not until 2012 that net public investments stabilised, albeit at a negative level of around -0.5% of GDP. This can be interpreted in the sense that the public capital stock in the periphery has been shrinking since then.

The euro area was hard hit by the coronavirus shock, with all indicators hinting at a record recession in 2020. According to the European Commission Spring forecast, the average GDP level of the periphery countries Italy, Spain, Portugal and Greece will be driven back below its 2007 pre-crisis level at least until 2021 after they had slowly recovered from the austerity crisis to that level in 2019 (Figure 3). Obviously, neither the euro area nor the crisis countries can afford a return to strict fiscal tightening or austerity. However, as can be seen in Figure 1, headline fiscal deficits in the periphery can be expected to be around 4% of GDP in 2021 and structural deficits between 2% to 3% of GDP with a very uncertain outlook for the years ahead. Once the general escape clause of the SGP is given up, many euro area countries will find themselves in trouble with the preventive arm of the SGP where structural deficits have to be substantially reduced to reach the medium term objective (MTO), if they do not find themselves in an excessive deficit procedure. Worse still, public debt levels in the periphery must be expected to increase strongly by 15 to 20 percentage points compared to 2019 (Figure 3), which could lead to stricter MTOs or an excessive deficit procedure; this, in turn, would lead to even stronger consolidation requirements. The resulting fiscal contraction could well ruin the prospects of recovery and medium term growth. However, it is clear that a return of austerity policies would not be sustainable economically, socially or politically. The recovery plan approved by the extraordinary Council meeting will alleviate the problems substantially in the short run, but it does not remove the necessity to reform fiscal rules to prevent further austerity over the medium term.

Figure 3

**General government debt-to-GDP ratio and real GDP level (2007=100), EMU and EMU periphery, 2007-2021**

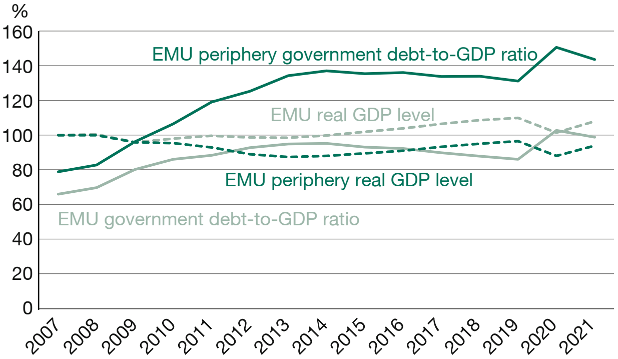

Note: The EMU periphery refers to Greece, Italy, Portugal and Spain, unweighted arithmetic average.

Source: European Commission (2020); author's calculations.

## More cyclical leeway for fiscal policy

So what direction should reform of the fiscal rules take? The first key element would be to significantly increase the cyclical flexibility of the SGP. One very pragmatic reform option which could be realised purely technically without amending the EU treaties would be to reconsider the EU Commission's method of cyclical adjustment, which plays an important role in budgetary surveillance. A major reason for the lack of economic adequacy of the SGP is that the procedures used for cyclical adjustment – though useful in principle – function only imperfectly. The structural budget balance is a fundamental factor in assessing national fiscal policy. Its development is used within the preventive arm of the SGP to assess whether member states have already achieved their MTO or whether they are taking adequate consolidation measures to achieve it. The change in the structural budget balance is also used as part of the excessive deficit procedure (exceeding the 3% limit) to assess whether progress towards consolidation is sufficient.

It is now widely accepted, however, that the change in the structural balance is a problematic indicator for the orientation of fiscal policy because it considerably underestimates the extent of fiscal restraint in phases of crisis and overestimates the success of consolidation during an upswing vice versa (see also Brooks and Fortun, 2020). The structural balance is calculated by cyclically adjusting the actual budget balance and correcting it for one-off effects (privatisation revenues, etc.). The usual cyclical adjustment methods underestimate the extent of cyclical fluctuations and lead to procyclical policies if they are used as a yardstick for fiscal policy. The EU Commission's method in particular has proven to be problematic because the calculated potential output is strongly influenced by the current economic situation. In phases of economic downturns, for example, potential output is quickly and sharply revised downwards, although this does not reflect real conditions (Truger, 2015a, 2016).

The downward revision of potential output has severe consequences for the calculated structural deficits and the consolidation efforts identified correspondingly. Consolidation efforts are usually estimated to be much lower than they actually were because a major part of the deficit is considered to be structural, although it may only have been caused by the economic downturn.[2] The easiest option would be to use medium-term averages for potential growth or, even better, to revise potential output estimates only in the medium term, e.g. every five years and not two or three times a year as is the case today. Such a potential calculation that is less sensitive to cyclical fluctuations, and which would have suspended the potential adjustment from spring 2010 onwards, would have opened up considerable room for manoeuvre for all member states under the preventive arm of the SGP (Truger, 2016). Italy, Luxembourg, the Netherlands, Austria and Finland would have reached their medium-term budgetary targets already by 2015 and would have had room for expansionary measures. Finally, the strongly negative output gaps would also have indicated an urgent need for fiscal action for the countries under the excessive deficit procedure. For the euro area as a whole, the output gap would have been -6.7% instead of -1.7% of GDP. This would have made it easy to justify the use of the exceptions to the SGP and would have made fiscal policy much less restrictive, even before 2015. Conversely, during recovery, the consolidation would automatically be greater and faster.

There are additional pragmatic changes that would simply make the fiscal leeway already expanded by the Commission (European Commission, 2015) even more flexible. As a matter of fact, following the changes to the decision-making rules for the deficit procedure by the six-pack and the Fiscal Compact, the Commission had considerable room to manoeuvre in decision-making (Seikel, 2016), which it already used in the cases of France, Spain and Portugal. A more far-reaching interpretation of the room to manoeuvre within the existing fiscal policy framework would be a seamless continuation of the EU Commission's new interpretation (Seikel and Truger, 2019).

More far-reaching, and well-justified, reforms could be to adjust the numerical limits in the SGP to more feasible levels. In order to avoid member states with predictably high levels of post-crisis debt being plunged immediately into counterproductive austerity, the SGP government debt limit would have to be raised significantly, for example, to 90% of GDP. The current limit of 60% was merely the EU average at the time the Maastricht criteria were adopted and is in no sense an evidence-based critical threshold. Such a move could not be expected to impair debt sustainability, especially in a situation where interest rates are low and can be expected to remain so. In line with a higher debt limit, the deficit variables of the SGP, the 3% deficit limit and the MTO, which ultimately correspond directly or indirectly with it, could also be adjusted upwards in order to provide substantially larger leeway for fiscal policy.

## Investment orientation: Implementing the golden rule

It would be even more important to reform the SGP so as to encourage public investment. As demonstrated in the EMU austerity crisis since 2010, public investment has been frequently cut hard during consolidation phases, as one of

2 The EU Commission has already acknowledged this problem (Carnot and Castro, 2015). It has revised the adjustment method several times (Hristov et al., 2017) and is now using additional indicators based on expenditure growth. This, however, considerably increases complexity and hardly alleviates the basic problem.

the few areas where economies can be realised rapidly. A reformed SGP should therefore strengthen public investment and protect it from crisis-driven cuts. As a pragmatic way to deal with public investment that is quite compatible with the current SGP framework, a golden rule for public investment should be introduced (see, for example, Truger, 2015b). This widely accepted traditional public finance concept would exclude net public investment from both the calculation of the headline and the structural deficit, so that net public investment is financed via deficits. Privileging public investment makes sense from an economic point of view. The golden rule strives for an intertemporal realisation of the pay-as-you-use principle in the case that present government spending provides future benefits. It allows financing of such spending (net public investment) by government deficits, thereby promoting intergenerational equity. Net public investment increases the public and/or social capital stock and provides benefits for future generations. Therefore, it is justified that future generations contribute to financing those investments via debt servicing. Future generations inherit the burden of public debt, but in exchange, they receive a corresponding public and/or social capital stock. Failure to allow for debt financing of future generations' benefits will lead to a disproportionate burden for the present generation through higher taxes or lower spending, creating incentives for the under-provision of public investment to the detriment of future generations.

While the exact definition of public investment must be discussed, a natural starting point for the analysis would be the classification in the national accounts, as it has received the most attention in the literature on growth effects. Bom and Ligthart (2014) conducted meta-regressions for the public capital-growth nexus. According to their results, the implied marginal returns are in the range of 10% (short run, national, all public capital) to 34.6% (long run, regional, core infrastructure). One may safely assume that traditional public investment has markedly positive growth effects. In addition to the longer-run supply-side effects, the short-run demand-side effects of public investment are also very favourable. In addition to its long-term economic advantages, it can be seen as the most effective short-run fiscal policy instrument (see, for example, Gechert, 2015).

The golden rule approach could also be combined with an expenditure rule concept abandoning the contested concept of structural deficit within the SGP (Alvarez et al., 2019; Dullien et al., 2020). Public investment should be favoured by separating current and investment budgets and submitting only the current budget to limits for nominal expenditure growth. The limits should be determined by the medium-term growth rate of real potential output plus the ECB target inflation rate of 2%. Increases in permanent nominal expenditure growth above this limit – which can be necessary to tackle social and ecological challenges – are then allowed if revenues are increased correspondingly. In case of general tax cuts, which are not recommended, expenditure growth rates should incorporate these withdrawals of resources. Using medium-term potential growth rates and the target inflation rate stabilises expenditure growth over the cycle and enables full working of automatic stabilisers.

### Welfare orientation and democratisation

Beyond the rather technocratic proposals for a reform of the fiscal rules, fiscal policy ultimately needs a reorientation, replacing technocratic limits on deficits and debt with the top-line goal of sustainable economic and social well-being (Alvarez et al., 2019, 20-25). Fiscal policy should be part of a broader multi-level governance framework aiming, for example, at the 'well-being of its peoples' or sustainable 'economic and social progress'. Both principles are also expressed in the European Treaties (see Feigl, 2017), in the overall global United Nations priorities provided by the Agenda for Sustainable Development (2015) and by Stiglitz et al. (2018).

In order to achieve sustainable and upward-convergent well-being, the EMU needs policy coordination well beyond numerical fiscal targets. The necessary coordination between member states could target material well-being, full employment, quality of life, ecological and economic sustainability. Countries should then follow an economic policy strategy that allows them to meet commonly agreed economic, social and environmental targets (see Mersch, 2018). One achievable proposal in this direction is to develop an integrated scoreboard of economic, social and environmental indicators to monitor developments and draw attention to deviations, which should be addressed by coordinated policies. They could be analysed in an annual well-being and convergence survey (AWCS; Feigl, 2017, 4) and broadly discussed at the beginning of the European Semester before drafting the priorities, at least within the European Parliament, the European Economic and Social Committee and the Macroeconomic Dialogue. The AWCS could be written by a council of economic, social and environmental experts, nominated, for example, by the European Parliament together with the European Economic and Social Committee. This council of experts should identify developments in well-being, convergence and sustainability and qualitatively assess targets, indicators and the current situation.

In order to increase transparency, accountability and democratic participation in EU decisions about fiscal policy and to make sure that the aforementioned results of the AWCS receive proper attention, one proposal would state that the positions taken by each minister within the Eurogroup should be made public so that a qualified discussion could take place at the national level. A second proposal would address more co-decision-making by the European Parliament, which should be expanded with regard to fiscal policy so that European citizens could really vote for certain policies and reject others. For that purpose, some kind of EMU parliament, for example as a subgroup of the European Parliament, could be created with the right to co-decide on all aspects of eurozone economic decision-making (see Andor et al., 2018).

The approval of the recovery plan for Europe by the extraordinary Council summit in July 2020 was a major breakthrough on the path to reforming EMU economic governance. The European Union should grasp this as an opportunity for deep reform of its dysfunctional fiscal rules.

Achim Truger, University of Duisburg-Essen; and German Council of Economic Experts, Wiesbaden, Germany.


References

Álvarez, N., G. Feigl, N. Koratzanis, M. Marterbauer, M. Mathieu, T. McDonnell, L. Pennacchi, C. Pierros, H. Sterdyniak, A. Truge and, J. Uxó (2019), Towards a Progressive EMU Economic Governance, ETUI Working Paper, 2019.13.
Andor, L., P. Berès, L. Bini Smaghi, L. Boone, S. Dullien, G. Duval, L. Garicano, M. A. Landesmann, G. Papaconstantinou, A. Roldan, G. Schick, X. Timbeau, A. Truger and S. Vallée (2018, 28 February), Blueprint for a Democratic Renewal of the Eurozone, Politico, https://www.politico.eu/article/opinion-blueprint-for-a-democratic-renewal-of-the-eurozone/ (15 September 2020).
Bom P Ligthart J What Have We Learned From Three Decades of Research on the Productivity of Public Capital? Journal of Economic Surveys 2014 28 5 889 916 10.1111/joes.12037
Brooks R Fortun J Eurozone Output Gaps and the COVID-19 Shock Intereconomics 2020 55 5 291 296
Carnot, N. and F. de Castro (2015), The Discretionary Fiscal Effort: An Assessment of Fiscal Policy and its Output Effect, European Commission Economic Papers, 543.
Dullien, S., C. Paetz, A. Watt and S. Watzka (2020), Proposals for a reform of the EU’s fiscal rules and economic governance, IMK Report, 159e.
European Commission (2015), Making the Best Use of the Flexibility within the Existing Rules of the Stability and Growth Pact, COM (2015) 12 final.
European Commission (2020), Annual Macro-Economic Database, Spring.
European Council (2015), Commonly Agreed Position on Flexibility in the Stability and Growth Pact, 14345/15 ECOFIN 888 UEM 422.
Feigl, G. (2017), From growth to well-being: a new paradigm for EU economic governance, ETUI Policy Brief, 2/2017.
Gechert S What fiscal policy is most effective? A meta-regression analysis Oxford Economic Papers 2015 67 3 553 580 10.1093/oep/gpv027
Hristov, A., C. Planas, W. Roeger and A. Rossi (2017), NAWRU Estimation Using Structural Labour Market Indicators, European Commission, European Economy Discussion Papers, 069.
Juncker, J.-C., D. Tusk, J. Dijsselbloem, M. Draghi and M. Schulz (2015), Completing Europe’s Economic and Monetary Union, Report, European Commission.
Mersch M. (ed.) (2018), Well-being for everyone in a sustainable Europe, Report of the independent commission for sustainable equality, https://www.progressivesociety.eu/publication/report-independent-commission-sustainable-equality-2019-2024.
Mourre, G., C. Astarita and S. Princen (2014), Adjusting the budget balance for the business cycle: the EU methodology, European Commission Economic Papers, 536.
Seikel D Flexible Austerity and Supranational Autonomy, The Reformed Excessive Deficit Procedure and the Asymmetry between Liberalization and Social Regulation in the EU Journal of Common Market Studies 2016 54 6 1398 1416 10.1111/jcms.12439
Seikel, D. and A. Truger (2019), The blocked completion of the European Monetary Union: Making the case for a pragmatic use of fiscal leeway, WSI Report, 52.
Stiglitz J. E., J.-P. Fitoussi and M. Durand (2018), Beyond GDP: Measuring what counts for economic and social performance, OECD Publishing.
Truger A Austerity, cyclical adjustment and the remaining leeway for expansionary fiscal policies within the current EU fiscal framework Journal for a Progressive Economy 2015 6 32 37
Truger, A. (2015b), Implementing the Golden Rule for Public Investment in Europe- Safeguarding Public Investment and Supporting the Recovery, Materialien zu Wirtschaft und Gesellschaft, 138.
Truger A Austeritätspolitik und Bildungskürzungen: Zur Diagnose und Therapie einer europäischen Krankheit, in L. Bellmann, G. Grözinger, W. Matiaske (eds.), Bildung in der Wissensgesellschaft Jahrbuch Ökonomie und Gesellschaft 2016 28 153 173
United Nations (2015), Transforming our world: the 2030 agenda for sustainable development, Resolution adopted by the general assembly on 25 September 2015, https://sustainabledevelopment.un.org/post2015/transformingourworld (15 September 2020).
